# Predictive imaging biomarkers on whole-body diffusion-weighted MRI (WB-DWMRI) and [^68^Ga]GaPSMA-PET/CT for [^177^Lu]LuPSMA therapy in metastatic prostate cancer (mCRPC)

**DOI:** 10.1186/s40644-026-01066-0

**Published:** 2026-06-30

**Authors:** Minal Padden-Modi, Jan Taprogge, Peter Dutey-Magni, Carolina G. S. Cauduro, Matthew D. Blackledge, Hoda Abdel-Aty, Iain Murray, Nabil Hujairi, Nina Tunariu, Nick James

**Affiliations:** 1 Radiotherapy and Imaging Department, Institute of Cancer Research, The Institute of Cancer Research, 123 Old Brompton Rd, South Kensington, London, SW7 3RP UK; 2 Radioisotope Physics, The Royal Marsden Hospital NHSFT & The Institute of Cancer Research, 15 Cotswold Rd, Sutton, London SM2 5NG UK; 3 Medicines Research Council, Clinical Trials Unit, University College London, 90 High Holborn, London, WC1V 6LJ UK; 4 Radiology Department, The Royal Marsden Hospital NHS FT, Downs Road, Sutton, Surrey SM2 5PT UK; 5 The Royal Marsden Hospital NHS FT, Fulham Road, London, SW36JJ UK

**Keywords:** [^177^Lu]Lu-PSMA radiopharmaceutical therapy, Prostate cancer, MRI, PSMA/PET CT, Predictive

## Abstract

**Background:**

This study evaluates the utility of quantitative imaging biomarkers derived from whole-body diffusion-weighted MRI (WB-DWMRI) and [^68^Ga]GaPSMA-PET/CT in predicting lesion-level response to [^177^Lu]LuPSMA therapy in metastatic castration-resistant prostate cancer (mCRPC).

**Methods:**

Twenty-two patients with mCRPC who underwent WB-DWMRI and [^68^Ga]GaPSMA-PET/CT within three months before [^177^Lu]LuPSMA therapy were identified. The PSMA SUV (SUV_mean_, SUV_SD_, SUV_peak_) and corresponding diffusion weighted imaging (DWI) parameters (ADC_mean_, ADC_kurtosis_, ADC_vol_) were extracted from five hottest lesions (highest SUV_mean_) on PSMA-PET/CT. Lesion response was assessed using modified PERCIST and MET-RADS-P criteria. Multilevel logistic regression and area under receiver operating characteristic (AUROC) analyses identified predictive biomarkers.

**Results:**

65 bone lesions and 30 lymph nodes were analysed pre- and post-therapy. Forty-four (68%) bone and 16 (53%) lymph nodes lesions responded to treatment. SUV_mean_ and SUV_peak_ were almost identical (rank-correlation = 0.97) and had high predictive performance for response with AUROC of 0.74 (95% CI: 0.62–0.86) and 0.75 (95% CI: 0.61–0.88) respectively. Lesion volume showed good performance (AUROC = 0.69, 95% CI 0.57–0.82) and moderately correlated with SUV_mean_ (rank-correlation = 0.43). Neither ADC_mean_ (AUROC = 0.45, *p* = 0.47) or ADC_kurtosis_ (AUROC = 0.49, *p* = 0.171) were predictive. On regression modelling, a 1-unit volume increase, raised response odds by 4.3 (95% CI 0.7–27) in bone lesions, and 6.2 (95% CI 0.6–67) in lymph nodes (*p* = 0.06). A 1-unit SUV_mean_ increase raised response odds by 1.5 (95% CI 1.1–2.0) in bone lesions and 1.2 (95% CI 1.0–1.4) in lymph nodes (*p* < 0.01). No evidence suggested combining volume with SUVmean enhanced predictive performance over SUV_mean_ alone (*p* = 0.58).

**Conclusions:**

Baseline PSMA SUV_mean_, SUV_peak_, and volume are promising predictive biomarkers for lesion-level response to [^177^Lu]LuPSMA therapy. Combining functional and structural imaging biomarkers could improve treatment stratification and response assessment. Further validation in larger studies, alongside patient-level analysis, and expansion beyond the five lesions is needed to refine predictive models for clinical application.

**Supplementary Information:**

The online version contains supplementary material available at 10.1186/s40644-026-01066-0.

## Background

Prostate cancer remains a major global health burden, with more than 1.46 million new cases and nearly 400,000 deaths annually [1]. Metastatic castration-resistant prostate cancer (mCRPC) represents its most lethal phenotype, defined by disease progression despite castration-level testosterone and associated with median overall survival (OS) of approximately 27 months [2]. Management of mCRPC has long relied on androgen-receptor pathway inhibitors (ARPIs) and taxane chemotherapy [3]. In contemporary practice, however, most patients reaching mCRPC stage have already received at least one ARPI +- taxanes, resulting in a population that is heavily pretreated. Although these agents offer meaningful benefit, responses are often limited and resistance is common [4, 5]. While effective for a small subset of disease, the limited reach of genotype-directed therapy highlights the need for treatments that are beneficial to a broader proportion of the mCRPC population [6].

Radioligand therapies targeting prostate-specific membrane antigen (PSMA) have emerged as a compelling solution [7, 8]. PSMA is highly expressed on most prostate cancer cells, making it an attractive target for phenotype-directed treatment [9]. [^177^Lu]LuPSMA radioligand therapy utilises this approach by coupling a PSMA-binding ligand with a ß-emitting radionuclide, enabling systemic delivery of targeted radiation to PSMA-expressing disease. The phase 3 VISION trial, which required PSMA‑positive positron emission tomography/computed tomography (PET/CT) for enrolment, confirmed the clinical impact of [^177^Lu]LuPSMA therapy by demonstrating significant improvements in progression‑free survival (PFS) and OS, leading to its widespread adoption. Yet treatment responses remain markedly heterogeneous [8]. In VISION, only ~ 46% of patients achieved a PSA50 decline, and only about 30% demonstrated a complete or partial radiological response per RECIST criteria, indicating that a substantial proportion of patients derive limited benefit despite meeting PSMA‑positivity criteria [8]. Therefore, identifying predictive imaging biomarkers is increasingly critical for stratifying which patients benefit most.

Both whole-body diffusion-weighted magnetic resonance imaging (WB-DWMRI) and [^68^Ga]GaPSMA PET/CT have demonstrated superior accuracy and sensitivity for metastatic disease detection compared to conventional imaging [10–12]. Importantly, these modalities provide semi-quantitative parameters such as standardised uptake value (SUV) from PSMA-PET/CT and the apparent diffusion coefficient (ADC) from WB-DWMRI, enabling detailed tumour characterisation. These metrics may aid pre-treatment patient stratification, disease assessment, and monitoring therapeutic response. WB-DWI MRI is now standard practise for patients with suspect metastatic disease within our institution. It is both non-ionizing, which allows for serial follow-up of changes following treatment, and characterises the cellular nature of the tumour prior to treatment via ADC quantification. It is always combined with whole-body fat/water Dixon imaging to identify areas of fatty displacement that are likely evident regions of metastatic disease. It provides complimentary information to PET as it offers direct visualization of tumour extent as it does not rely on radiotracer uptake.

High PSMA uptake on PSMA-PET/CT has become an established selection criteria for [^177^Lu]LuPSMA therapy [13]. SUV has been shown to correlate with absorbed radiation doses and radiological response in both mCRPC [14] and metastatic hormone-sensitive prostate cancer (mHSPC) [15, 16]. Beyond SUV, baseline PSMA-PET/CT quantitative biomarkers such as PSMA-positive tumour volume (PSMA‑TV) and total lesion PSMA (TL‑PSMA/TLP; volume-weighted uptake burden) have been associated with outcomes after [^177^Lu]LuPSMA therapy and are increasingly used as potential stratification variables [16–19]. In lesion-level analyses, baseline uptake intensity and relative uptake measures (e.g., SUVmax, SUVpeak, SUVmean) also show discriminatory ability for subsequent PET-defined lesion response/progression [16, 18, 20, 21]. Finally, uptake heterogeneity has been explored to capture inter-/intra-lesional variability and improve response prediction beyond conventional metrics [22–24].

Quantitative WB-MRI biomarkers, especially DWI/ADC-derived metrics, are biologically attractive in the [^177^Lu]LuPSMA setting because they characterise tumour cellularity and diffusion-restricted burden independent of PSMA expression, yet there remains little published work validating pretreatment ADC metrics as predictors of response after PSMA radioligand therapy [10]. As a result, much of the motivation for ADC-based feature extraction is extrapolated from other marrow-predominant malignancies, most prominently multiple myeloma, where response assessment systems and quantitative approaches using ADC metrics and diffusion-defined volumetrics have been linked to clinical outcomes [25, 26]. Importantly, the few studies that incorporate MRI alongside PSMA-PET/CT in the mCRPC for radioligand therapy response prediction remain limited. Roll et al. performed pretherapeutic PSMA-PET/MRI radiomics and reported MRI-derived predictors (primarily from T1/T2-weighted sequences mapped to PET-positive volumes) for biochemical response and survival, but did not evaluate DWI/ADC metrics. Several studies demonstrated an inverse correlation between baseline DWMRI ADC characteristics and Gleason grade in local PCa. However, no such study exists for mCRPC [27–29]. This highlights both the feasibility of multimodal imaging biomarker extraction and the persistent gap in ADC-focused work in this treatment context [30].

Prognosis and therapeutic outcomes in mCRPC also vary based on disease site. Halabi et al. demonstrated OS varied according to metastatic site; lymph-node-only disease (31.6 months) versus bone-metastases-only (21.3 months) [31, 32], highlighting the importance of accounting for distinct biological and clinical behaviours of metastases at different sites.

With PET‑based predictors well established but quantitative WB‑MRI evidence still limited, we aimed to test whether both modalities contribute complementary predictive value while also statistically accounting for the sites of disease. We evaluated whether SUV features from PSMA-PET/CT and ADC features from WB-DWMRI predict treatment response to [^177^Lu]LuPSMA at the individual‑metastasis level.

Materials and Methods.

### Study participants

Patients who underwent a WB-DWMRI and [^68^Ga]GaPSMA-PET/CT imaging prior to [^177^Lu]LuPSMA therapy were identified from a high-volume regional institution imaging database. Data was retrospectively collected and included any patients scanned between June 2019 and January 2023. The study was registered as a formal service evaluation following approval from the Institutional Review Board. As the study was retrospective, the need to obtain consent was waived.

The inclusion criteria for patient eligibility were:

- Diagnosis of mCRPC.
- Treated with ≥ 1 cycle of [^177^Lu]LuPSMA therapy.
- Baseline WB-DWMRI and [^68^Ga]GaPSMA-PET/CT performed within three months of initiating radiopharmaceutical therapy (RPT).
- Post-treatment imaging assessment with [^68^Ga]GaPSMA-PET/CT or WB-DWMRI within 6 months after treatment.

Patients were excluded if they underwent alternate therapies between imaging and [^177^Lu]LuPSMA therapy initiation.

### Imaging assessments and quantitative imaging metrics

[^68^Ga]GaPSMA-11 PET images were acquired on Biograph mCT or Biograph Horizon PET/CT scanners (Siemens Healthineers, Germany). Patients were injected with nominally 150 MBq of [^68^Ga]GaPSMA-11 and images were acquired 60 min after administration for 3 min/bed or with a speed of 0.7 mm/s, respectively. Low dose CT images were reconstructed at 3 mm slice thickness and separation. Images were reconstructed using an iterative OSEM algorithm incorporating time of flight measurement with slice thickness matched to the CT data.

All DWI images were acquired using a standard institutional sequence using a 1.5T scanner (Magnetom, Siemens Healthineers, Germany) using three b-values (50, 600, and 900 s/mm^2^), short-tau inversion recovery fat suppression (inversion time = 180ms), with a bipolar diffusion weighted acquisition scheme to supress eddy current distortions. Images were acquired in contiguous stations, each comprising 40 slices with a slice thickness of 5 mm and in-plane pixel spacing of approximately 1.6 mm following interpolation.

We used quantitative imaging software, HERMES HERMIA Hybrid Viewer v7.0.2 (Hermes Medical Solutions AB, Stockholm) to delineate the 5 lesions with the highest SUV_mean_ on [^68^Ga]GaPSMA-PET/CT indicating the highest expression of PSMA (‘hottest lesions’) per patient as a pragmatic approach, which also aligned with guidance outlined by PERCIST1.1 [33]. Disease was defined as lesions exhibiting an SUV exceeding the liver-threshold SUV. A 3 cm spherical region was applied to the normal liver to calculate the SUV-threshold, for standardised lesion quantification. This follows established quantitative PSMA (qPSMA) methods [34] using the formula:

$$\eqalign{ {\rm{SU}}{{\rm{V}}_{{\rm{threshold}}}}{\rm{ = }} & {{{\rm{4}}{\rm{.30}}} \over {{\rm{SU}}{{\rm{V}}_{{\rm{mean}}}}}} \cr & {\rm{ \times }}\left( {{\rm{SU}}{{\rm{V}}_{{\rm{mean}}}}{\rm{ + SU}}{{\rm{V}}_{{\rm{SD}}}}} \right) \cr}$$

SUV_threshold_: Threshold SUV applied to standardise lesion delineation.

SU_mean_: The mean SUV of the 3 cm liver sphere.

SUV_SD_: The Standard Deviation (SD) of the SUV_mean_ in the 3 cm liver sphere.

For each patient, once the five “hottest” lesions were selected, corresponding lesions were identified and delineated on the B900 sequence of WB-DWMRI, and their ADC characteristics extracted using PyOsirix v0.2.1b6 (OsiriX, Pixmeo, Geneva). Bone lesions were identified using the fat-fraction image for reference, and soft tissue lesions were delineated with reference to the T2 signal image to align anatomical boundaries across MRI and PET/CT. Lesions were contoured by a clinical research fellow and corrected and verified by a specialist MRI radiologist. The following SUV and ADC metrics (for details see Supplementary Table S1) were extracted at baseline and post-treatment for each target lesion: SUV_mean_, SUV_SD_, SUV_peak_, ADC_mean_, ADC_SD_, ADC_kurtosis_ and ADC_vol_.

SUV metrics directly reflect uptake of a ligand within target tissue and therefore should be predictive of therapeutic ligand uptake. SUV_peak_ was selected in preference to SUV_max_ due to its relative statistical robustness. Although WB-DWMRI derived metrics do not directly reflect ligand uptake, ADC_mean_ is a commonly quoted parameter within literature to characterize the overall cellularity within tumours [35]. However, it is known that tumour exhibit distinct heterogenous imaging phenotypes and so our purpose for ADC_SD_ and ADC_kurtosis_ was to explore this inherent heterogeneity but understanding the spread in ADC estimates and the deviation of the ADC histogram from a normal distribution. All derived ADC metrics have been shown to have modest to good reproducibility in whole-body imaging, as discussed in a prior publication [36].

### Response assessment

Either [^68^Ga]GaPSMA-PET/CT or WB-DWMRI were used as response assessments. PERCIST criteria [33] was used for PSMA-PET/CT and MET-RADS-P [37] for WB-DWMRI. Both were modified to assess lesion-level response as opposed to patient-level response (Table 1).

**Table 1:** Lesion-level response assessment criteria

Response Assessment	PSMA-PET/CT (Modified PERCIST 1.1)	WB-DWMRI (Modified MET-RADS-*P*)
Progressive Disease (PD)	SUV_peak_ ↑ ≥30% and absolute ↑ ≥0.8 units.	Volume ↑ >20% with ADC 600–1000 μm²/s.
Stable Disease (SD)	Between PD and Response.	Between PD and Response.
Response (R)	SUV_peak_ ↓ ≥30% and absolute ↓ ≥0.8 units.	ADC ↑ >25% +/- volume ↓ >30%.

Key: ↑: increase, ↓: decrease

### Statistical analysis

Statistical analysis was performed using Stata (Stata18, StataCorp, Texas). Descriptive statistics, including point and interval mean estimates were calculated for baseline values and change scores. Spearman’s rank correlation coefficients were calculated between baseline imaging metrics. To account for statistical dependence of measurements within patients, robust Huber-White standard errors were used.

The predictive performance of each metric (SUV and ADC) for lesion-level response, was evaluated using the full area under the receiver operating characteristic curve (AUROC). AUROC is a measure of diagnostic performance ranging between 0.5 (no predictive power) and 1 (perfect predictive power). AUROC estimates were produced under a cluster bootstrap (1000 replications) to account for the statistical dependence between lesions belonging to the same patient.

Multilevel logistic regression models were used to study the relationship between the three most predictive quantitative metrics and the odds of lesional response. To address the strong right-skew in ADC_vol_, log10 transformation was applied. Each metric was centred around the global mean to facilitate model fitting. Second-degree fractional polynomial transformations were assessed to model potentially non-linear relationships, with the optimal transformation selected using sequential likelihood ratio tests (significance threshold: *p* < 0.05) [38].

We compared models regressing lesion response on:

1. the effect of lesion type alone.
2. the interaction of lesion type and baseline SUV_mean_.
3. the interaction of lesion type and baseline ADC_mean_.
4. the interaction of lesion type and baseline ADC_vol_.
5. the interaction of lesion type, baseline ADC_mean_ and SUV_mean_.

The statistical significance of the association between the biomarker and odds of response was evaluated by comparing these nested models using a likelihood ratio test.

## Results

Twenty-two patients with mCRPC with baseline [^68^Ga]GaPSMA-PET/CT and WB-DWMRI were included in the study. Patients were predominantly in the 4th or higher treatment line and median PSA prior to [^177^Lu]LuPSMA therapy was 44.5 ng/mL (interquartile range (IQR):14–76). Complete baseline patient characteristics can be found in Table 2. Of the 22 patients, 20 had assessable lesions on post-treatment imaging, from which 95 lesions (65 bone, 30 lymph node) were analysed. At baseline, the mean SUV_mean_ was 15.0 (95% CI:12.9–17.1) across all lesions analysed. The mean SUV_mean_ for lymph node lesions was 12.5 (95% CI:8.6–16.4), and 16.2 for bone lesions (95% CI:13.7–18.7). Lesion descriptive characteristics at baseline are summarised in Table 3.

**Table 2:** Baseline patient characteristics

Baseline Patient Characteristics		
Age (years)	Median 75.5 (range:60–88; IQR:70–80)	
Gleason Score at Diagnosis	Median 9 (range:7–10; IQR:7–9)	
Stage at Diagnosis	localised disease	13
	metastatic disease	9
Prior Lines of Therapy	Median 3 (range:1–11; IQR:2-5.75)	
Pre-therapy PSA (ng/mL)	Median 44.5 (range:8.5–1107; IQR:14–76)	

IQR: interquartile range

**Table 3:** Baseline and response characteristics of lesions by type, with robust confidence interval estimates

	Lesion Type								
	Bone			Lymph Node			All		
	Mean	95% CI	*N*	Mean	95% CI	*N*	Mean	95% CI	*N*
Baseline									
SUV_mean_	16.2	13.7, 18.7	65	12.5	8.6, 16.4	30	15.0	12.9, 17.1	95
ADC_mean_	808.9	716.1, 901.7	65	1075.2	877.0, 1273.4	30	893.0	792.4, 993.6	95
ADC_vol_ (mL)	13.2	0.7, 25.7	65	2.1	1.0, 3.1	30	9.7	1.1, 18.2	95
log10 ADC_vol_ (mL)	0.6	0.3, 0.8	65	0.0	-0.2, 0.2	30	0.4	0.2, 0.6	95
**Lesion response category (%)**									
Progression	12.3	3.2, 37.2	8	13.3	2.9, 44.4	4	12.6	4.8, 29.2	12
Response	67.7	45.2, 84.2	44	53.3	23.0, 81.4	16	63.2	45.2, 78.1	60
Stable	20.0	10.3, 35.3	13	33.3	11.9, 64.9	10	24.2	13.8, 39.0	23

Note: robust confidence intervals limits estimated with cluster-robust Huber-White standard errors

CI: confidence intervals, N = number of lesions

### Lesion response

The majority of patients were followed up using [^68^Ga]GaPSMA-PET/CT imaging (*n* = 12) and 56 lesions were therefore classified using PERCIST. The remaining 39 lesions (*n* = 8 patients) were classified according to MET-RADS-P. Following [^177^Lu]LuPSMA therapy, 63% of lesions (60/95) responded, 24% (23/95) were stable, and 13% (12/95) progressed. A higher proportion of disease response was observed in bone lesions (67.7%, 95% CI: 45.2, 84.2) compared to lymph node lesions (53.3%, 95% CI: 23.0, 81.4). Full response characteristics identified by lesion site are detailed in Table 3.

### Correlation of imaging metrics

Spearman’s rank correlation coefficients between baseline imaging metrics are presented in Table 4. Unsurprisingly, SUV_mean_ and SUV_peak_ were highly correlated (ρ = 0.97, *p* < 0.05) which is why only SUV_mean_ was selected for regression modelling. ADC_mean_ showed weak or no correlation with SUV metrics, while ADC_vol_ correlated moderately with both SUV_mean_ (ρ = 0.43, *p* < 0.05) and SUV_peak_ (ρ = 0.51, *p* < 0.05).

**Table 4:** Pairwise Spearman’s rank correlations for baseline lesion characteristics

	SUVmean	SUVpeak	ADCmean	ADCkurt	ADCstd	ADCvol
SUV_mean_	1.00					
SUV_peak_	0.97*	1.00				
ADC_mean_	-0.07	-0.05	1.00			
ADC_kurtosis_	0.23*	0.24*	-0.16	1.00		
ADC_std_	0.01	0.08	0.45*	-0.03	1.00	
ADC_vol_	0.43*	0.51*	-0.10	0.37*	0.26*	1.00

Note: * indicates *p* < 0.05

### Predictive performance of imaging metrics

**Fig. 1:**
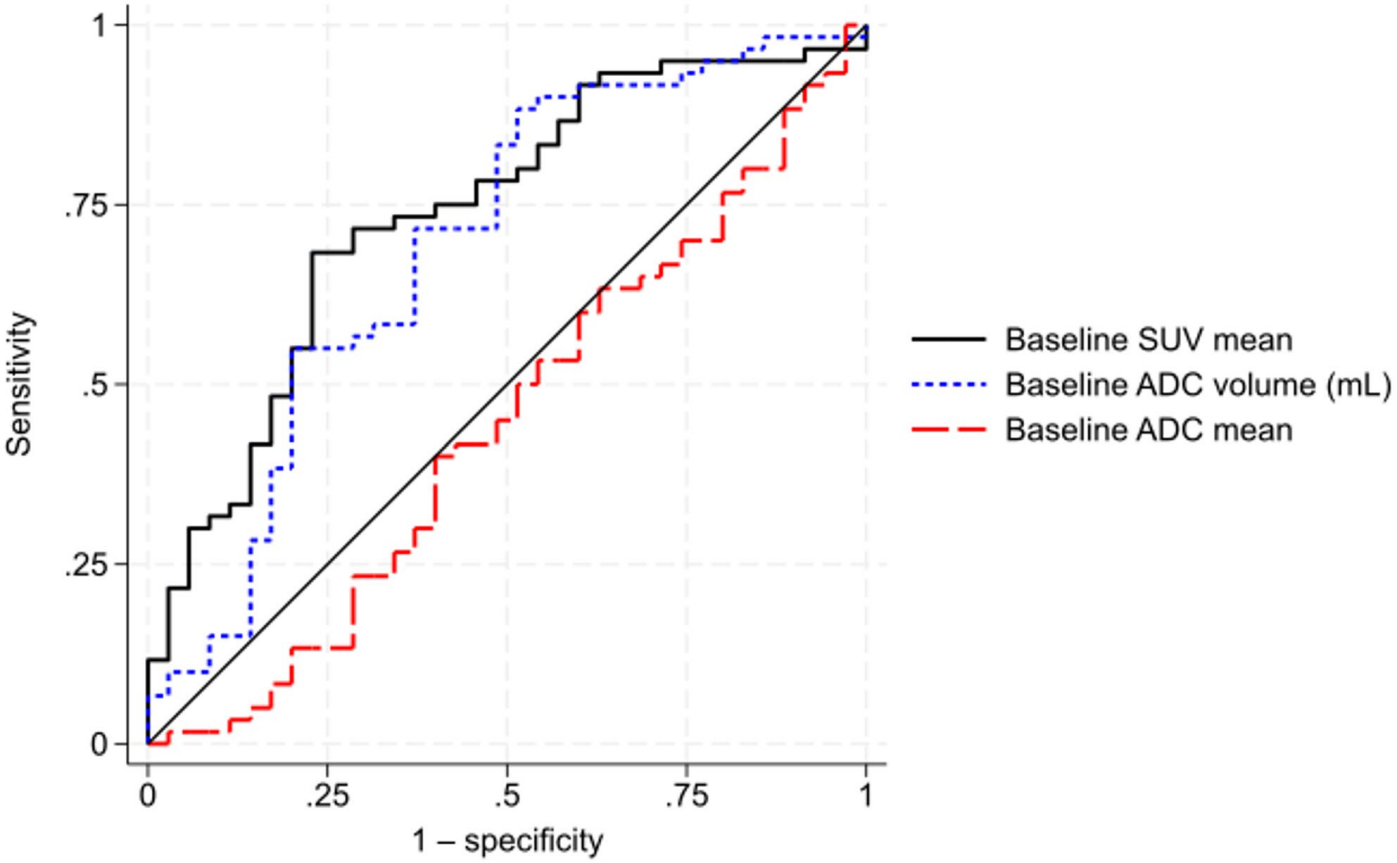
Receiver operating characteristics curves for SUVmean (black solid line), ADCmean (blue dashed line) and ADCvol (red dashed line) in predicting lesion response versus stable disease/progression

**Fig. 2:**
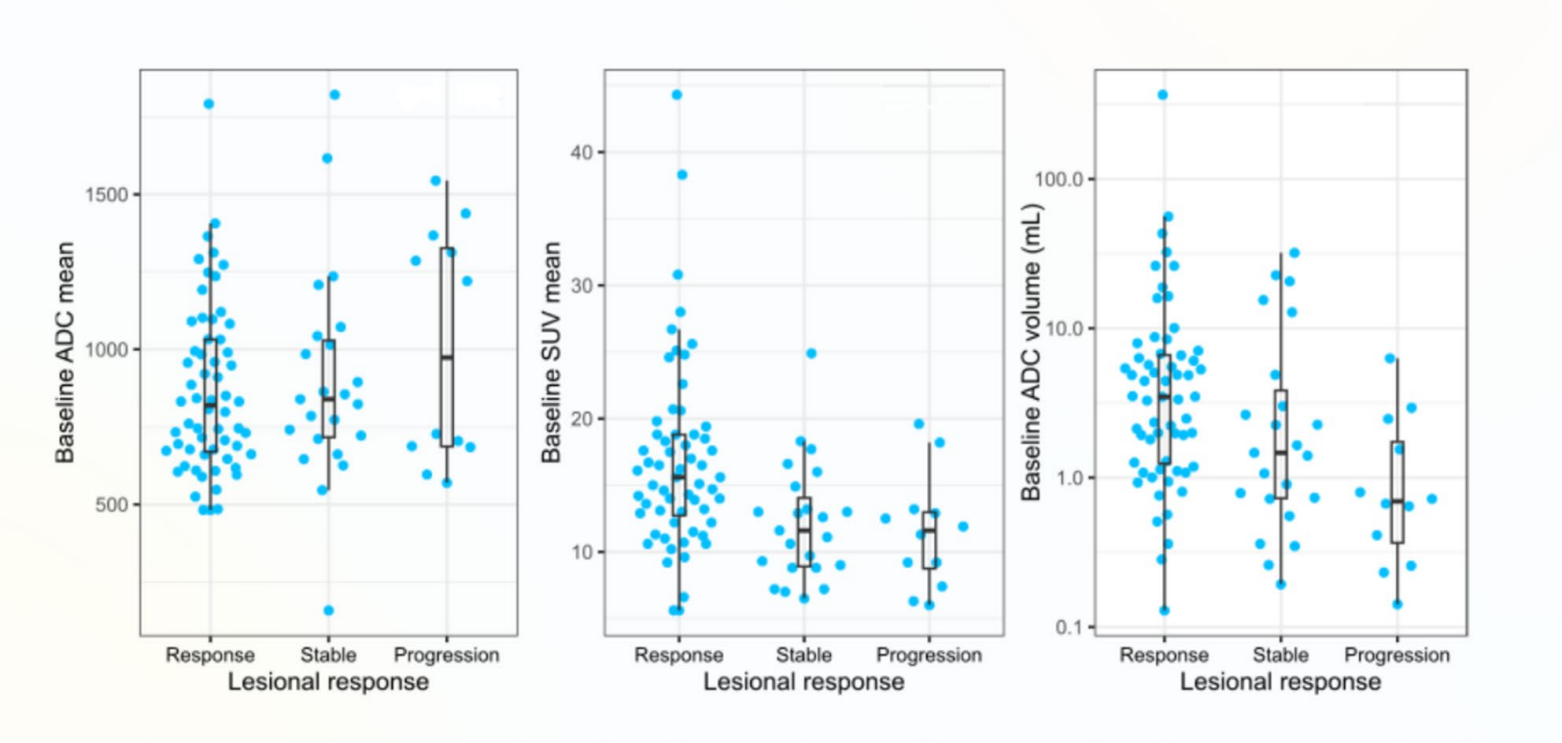
Graphs comparing the mean baseline imaging parameter between response, stable and progressive lesions for (**A**) ADC mean, (**B**) SUVmean, (**C**) ADC vol. Boxplots show the median and quartiles with the whiskers representing 1.5 times the IQR

The predictive performance of baseline metrics derived from [^68^Ga]GaPSMA-PET/CT and WB-DWMRI images were assessed using ROC analysis. Of the metrics tested, SUV_mean_ and SUV_peak_ had the highest AUROCs at 0.74 (95% CI:0.62–0.86) and 0.75 (95% CI:0.61–0.88), respectively. ADC_vol_ demonstrated moderate predictive performance (AUROC = 0.69, 95% CI:0.57–0.82). The other ADC metrics, ADC_mean_, ADC_sd_ and ADC_kurtosis_ did not perform better than chance (AUROC ≈ 0.5). A complete summary of these results is provided in Table 5, whilst Fig. 1 displays the ROC curves for SUV_mean_, ADC_mean_, and ADC_vol_. Figure 2 illustrates the differences in baseline, ADC_mean_, SUV_mean_ and ADC_vol_ between responding, stable, and progressing lesions.

**Table 5:** Non-parametric AUROC estimates for the diagnostic performance in predicting lesion response

	AUROC	95% CI	SE	*p*-value
SUV_mean_	0.74	0.62, 0.86	0.063	< 0.001
SUV_peak_	0.75	0.61, 0.88	0.069	< 0.001
ADC_mean_	0.45	0.29, 0.60	0.077	0.477
ADC_kurt_	0.59	0.46, 0.71	0.064	0.171
ADC_vol_	0.69	0.57, 0.82	0.065	0.003
ADC_std_	0.54	0.38, 0.71	0.085	0.606

Note: Estimates produced using a bias-corrected clustered bootstrap. The p-value tests the null hypothesis AUROC = 0.5

CI: confidence intervals, SE = standard error

### Regression modelling

**Fig. 3:**
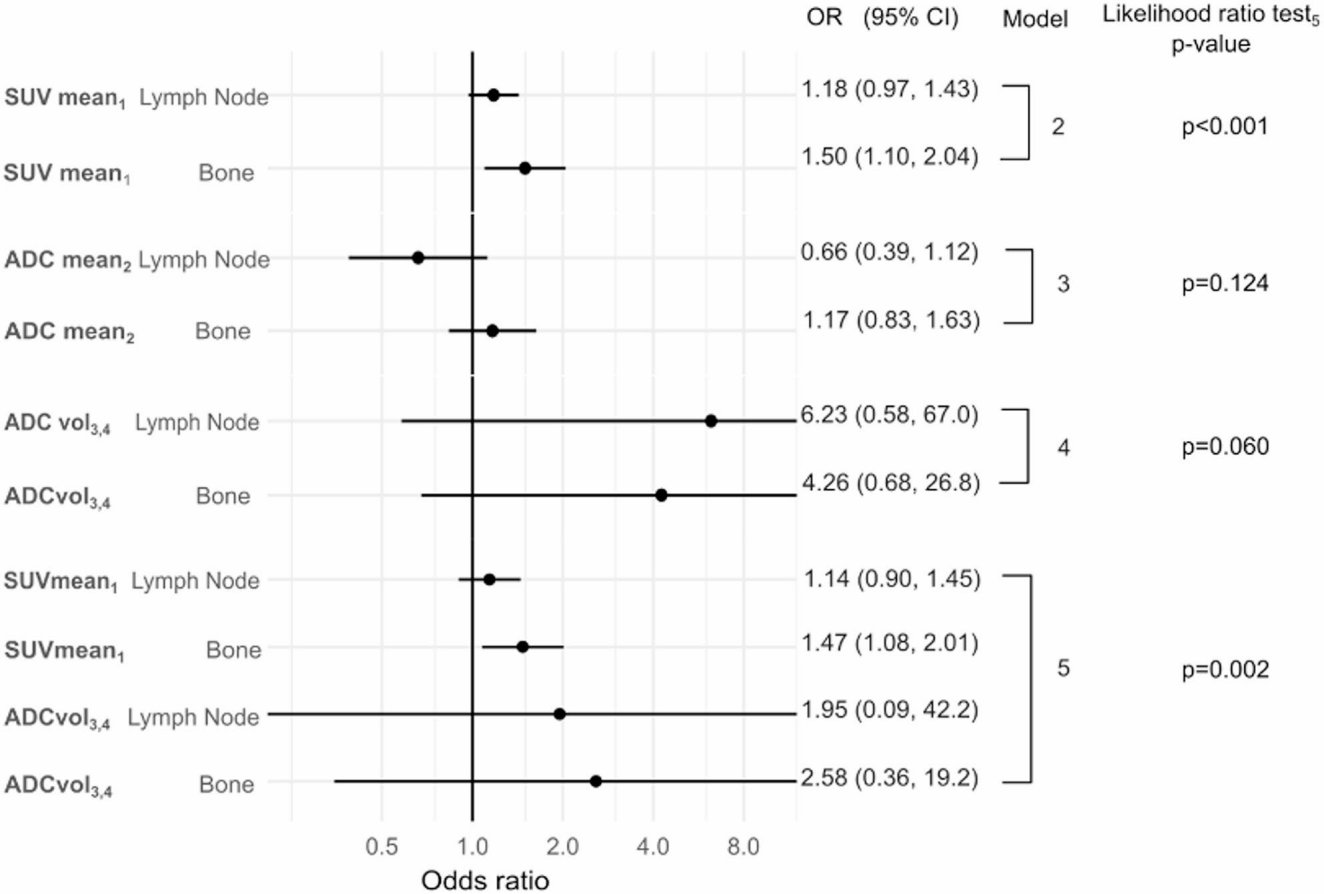
Forest plot of multilevel logistic regression coefficient

The association between baseline ADC_mean_, ADC_vol_ and SUV_mean_ metrics and the odds of lesion response was further analysed in multilevel logistic regression models (Fig. 3). All three metrics were modelled as continuous variables, after determining that no fractional polynomial transformation significantly improved the fit of the model. To assess the statistical significance of associations, each model was compared with a null Model 1 (Supplementary Table S2) containing only the lesion type variable. In this model, lymph node lesions had similar odds of response as bone lesions (OR = 0.97, 95% CI:0.15–6.37).

We found significant evidence of an association between baseline SUV_mean_ and lesion response (model 2): each 1-unit increase in SUV_mean_ increased the odds of lesion response by 1.5 (95% CI:1.1-2.0) for bone lesions and 1.2 (95% CI:1.0-1.4) for lymph node lesions.

DWI-derived ADC_vol_ (which was log10-transformed) was positively associated with odds of lesion response (model 4). Every 1-unit increase in ADC_vol_ multiplied the odds of lesion response by 4.3 (95% CI:0.7–27) and 6.2 (95% CI:0.6–67) in bone and lymph node lesions respectively. However, this model was not significantly better than a model with lesion type only (*p* = 0.060). In model 5, the addition of ADC_vol_ to the best fitting model 2 (SUV_mean_ by lesion type) also did not significantly improve the fit (*p* = 0.581). Finally, there was no evidence that ADC_mean_ improved the model fit (*p* = 0.124) and odds ratio coefficients equalled 1, suggesting no association.

## Discussion

This study evaluated whether baseline [^68^Ga]GaPSMA-PET/CT and WB-DWMRI metrics predict lesion-level imaging response after [^177^Lu]LuPSMA therapy in heavily pre-treated mCRPC. Lesion response was assessed using modified PERCIST 1.1 and modified MET-RADS-P (Table 1). To our knowledge, most prior predictive imaging work in this setting has been PET-only or has focused on patient-level outcomes rather than lesion-level imaging response, and WB-DWMRI has rarely been tested as a baseline predictor of lesion-response in [^177^Lu]LuPSMA cohorts.

Using bias-corrected clustered bootstrap ROC analysis to account for within-patient correlation, baseline PET uptake metrics demonstrated the strongest discrimination for lesion-level response. SUV_mean_ (AUROC 0.74, 95% CI 0.62–0.86) and SUVpeak (AUROC 0.75, 95% CI 0.61–0.88) both showed highest predictive performance. Among WB-DWMRI metrics, DWI derived-lesion volume (ADC_vol_) demonstrated moderate performance (AUROC 0.69, 95% CI 0.57–0.82), whereas ADC_mean_ was not predictive, and other ADC distribution metrics performed weakly (Fig. 1; Table 5).

Our findings are consistent with the lesion-level PET response literature, where baseline uptake intensity repeatedly emerges as a key predictor. Van der Sar et al. performed lesion and patient-level analyses and demonstrated that the baseline lesion SUV_peak_/SUV_max_ was strongly associated with lesion-level imaging response after two cycles of [^177^Lu]LuPSMA therapy [20]. Their conclusion, that baseline uptake intensity is more predictive of lesion response than baseline volumetric measures, aligns closely with our observation that SUV metrics outperformed ADC metrics and that ADC_vol_ did not significantly improve model fit beyond baseline SUV_mean_ (Fig. 3; Table 5) [20].

Similarly, Groener et al. analysed a large lesion dataset and found that baseline SUV_max_/SUV_mean_ (and tumour to liver ratio) could predict responding versus non-responding lesions [21]. The AUROC values observed in our study (~ 0.75) fall within a similar range as that in their study, reinforcing that quantitative SUV measures provide predictive information at lesion level. Bülbül et al. used baseline PET texture features combined with machine learning approaches and reported very high lesion level predictive performance (k nearest neighbours AUC: 0.95) [22]. While these results support the feasibility of lesion-level prediction, the substantially different methodology (radiomics/ML, small patient sample) warrants caution due to reduced reproducibility relative to conventional metrics.

Beyond lesion-level response studies, larger-scale evidence supports the clinical relevance of quantitative PSMA-PET/CT biomarkers. The quantitative PSMA-PET/CT analysis from the VISION trial demonstrated associations between baseline PSMA-PET/CT parameters and clinical outcomes in the context of a large multicentre trial [16]. In addition, a recent systematic review and meta-analysis concluded that quantitative PSMA-PET/CT biomarkers, including uptake and tumour burden measures, show consistent associations with treatment response and outcomes across studies, despite heterogeneity in endpoints and definitions [19]. Although these works are not lesion-level imaging-response prediction studies, they strengthen the interpretation that quantitative measures on baseline PSMA-PET/CT may provide clinically meaningful information and aid personalisation in our approach to treatment with [^177^Lu]LuPSMA therapy [16, 18, 19].

In contrast, ADC metrics from WB-DWMRI showed variable results. While DWI-derived volume (AUROC = 0.69) exhibited moderate predictive value, ADC_mean_ and ADC_kurtosis_ were not predictive of response on both multivariate (combined with SUV_mean_) and on univariate models (Tables 4 and 5; Fig. 1). Furthermore, although ADC_vol_ was found to a predictive marker by ROC analysis (Tables 4 and 5), the positive odds ratio associated with regression modelling of ADC_vol_ did not reach statistical significance (Fig. 3).

The lack of predictive value for WB-DWMRI metrics should be interpreted considering heterogeneous post‑treatment response assessments, with lesions evaluated using either PSMA-PET/CT or WB-DWMRI depending on clinical availability. The variable response assessments may dilute the contribution of MRI‑derived predictors, rather than indicating a true lack of relevant imaging signal. Accordingly, WB‑DWMRI should be viewed as a complementary, PSMA‑agnostic modality that captures tissue and disease characteristics distinct from PET uptake, rather than as a direct substitute for PET‑based metrics in baseline lesion‑level response prediction.

Placed within the broader literature, these findings help contextualise the role of MRI in the [^177^Lu]LuPSMA setting. To our knowledge, few studies have integrated MRI and PET information in patients receiving [^177^Lu]LuPSMA therapy, and existing work has primarily focused on radiomic analysis of baseline hybrid PET‑MRI rather than on WB‑DWMRI–derived quantitative metrics or lesion‑level imaging response. In a study by Roll et al., MRI‑derived radiomic features from T2‑weighted sequences showed predictive and prognostic associations with biochemical response and survival [30]. Together with the present results, this suggests that the contribution of WB-DWMRI in metastatic prostate cancer may depend on how information is extracted and analysed, and that simple summary metrics may be insufficient to capture clinically relevant features lesion level which could be predictive.

PSMA-PET/CT has limited value in detecting PSMA-negative prostate cancer potentially reducing its sensitivity in identifying metastases. Despite comparable histopathological features to PSMA-positive tumours [39, 40], PSMA-PET/CT negative disease represents 3–10% of primary prostate cancer [39–41] and was observed in 4.2% of mHSPC cases (UpfrontPSMA) [42] and 18% of mCRPC (TheraP) where [^18^F]FDG-PET/CT identified discordant disease [43].

Unlike PSMA-PET/CT, WB-DWMRI imaging does not rely on PSMA expression, and can detect PSMA-negative disease, likely resistant to targeted [^177^Lu]LuPSMA therapy. WB-DWMRI, while not predictive of lesion-level response in this study, may play a greater role in response assessment in mCRPC by independently evaluating disease cellularity and anatomy regardless of PSMA-expression.

Several limitations should be acknowledged. First, this retrospective study had a small heterogenous sample size (*N* = 22 patients, 95 lesions) limiting the generalisability and statistical power of our findings. Secondly, selecting only the five highest-uptake lesions per patient may have introduced selection bias, by excluding smaller or less active lesions that could influence clinical outcomes. Thirdly, while modified PERCIST 1.1 and MET-RADS-P criteria provided structured response assessment, their application at lesion-level warrants further validation. In addition, with this being a retrospective study, imaging criteria for response had to reflect the institutional practice leading to heterogeneity in post‑treatment response assessment. Lesions were evaluated using either PERCIST 1.1 for [^68^Ga]GaPSMA-PET/CT or MET-RADS-P for WB‑DWMRI depending on clinical availability, which may have potentially grouped lesions into different response outcomes. Finally, while lesion‑level response provides insight into intra‑patient heterogeneity, direct linkage between lesion‑specific imaging findings and patient‑level outcomes could not be explored in this cohort. These limitations highlight the need for larger, prospective studies with harmonised imaging protocols and response definitions.

Future research could validate our findings in larger, multicentre prospective cohorts with standardised imaging protocols and validated response assessments. Prospective studies could combine [^68^Ga]GaPSMA-PET/CT and WB-DWMRI with clinical, molecular, and histological parameters, to develop more predictive models, which could be validated through biopsy and biomarker studies. This approach could offer insights into tumour biology and therapy resistance mechanisms. Additionally, expanding analysis to post-therapy ^177^Lu-SPECT imaging and dosimetry [44–46], may further improve our understanding of response characteristics.

The STAMPEDE2 trial [47] is currently investigating upfront accelerated [^177^Lu]LuPSMA therapy in the metastatic hormone-sensitive prostate cancer setting and includes an imaging sub‑study with multi‑time‑point WB‑DWMRI, [^68^Ga]GaPSMA-PET/CT, and three‑time‑point SPECT acquisitions, enabling lesion‑level dosimetry across treatment cycles. Through this, our group is prospectively evaluating multimodality imaging and exploring integration with tissue biopsies and circulating tumour biomarkers, which may further complement imaging‑based predictors of treatment response.

## Conclusion

This study demonstrates that baseline [^68^Ga]GaPSMA‑PET/CT uptake metrics are informative at the lesion level for imaging response to [^177^Lu]LuPSMA therapy in heavily pre‑treated mCRPC, reinforcing the value of quantitative PET for capturing spatial heterogeneity of treatment effects. By focusing on individual metastases rather than aggregate patient‑level outcomes, our findings highlight the importance of lesion‑specific analysis in understanding response and resistance to radioligand therapy.

While WB‑DWMRI–derived metrics did not show a clear independent contribution to baseline lesion‑level response prediction in this cohort, the present results do not exclude a meaningful role for MRI‑based biomarkers. Instead, they underscore that the added value of MRI in this setting remains to be defined, and may depend on study scale, response definitions, and the way imaging features are extracted and integrated. Further prospective, adequately powered studies are therefore required to clarify how [^68^Ga]GaPSMA‑PET/CT and WB‑DWMRI can be optimally combined to refine patient and lesion‑level stratification in PSMA‑targeted radioligand therapy.

## Supplementary Information

Below is the link to the electronic supplementary material.

Supplementary Material 1

## Data Availability

All data is available from the corresponding author, Dr Minal Padden-Modi: (mailto: minal.padden-modi@icr.ac.uk). Dr Peter Dutey-Magni: (mailto: p.dutey-magni@ucl.ac.uk), who has significant statistical expertise, kindly provided statistical advice for this manuscript.

## Abbreviations

DWI: Diffusion weighted imaging
mCRPC: Metastatic castration-resistant prostate cancer
OS: Overall survival
ARPIs: Androgen-receptor pathway inhibitors
PSMA: Prostate-specific membrane antigen
PET/CT: Positron emission tomography/computed tomography
PFS: Progression‑free survival
WB-DWMRI: Whole-body diffusion-weighted magnetic resonance imaging
SUV: Standardised uptake value
ADC: Apparent diffusion coefficient
mHSPC: Metastatic hormone-sensitive prostate cancer
PSMA‑TV: PSMA-positive tumour volume
TL‑PSMA: Total lesion PSMA
AUROC: Area under the receiver operating characteristic curve
