# Supplementary material for: Predictive imaging biomarkers on whole-body diffusion-weighted MRI (WB-DWMRI) and [68Ga]GaPSMA-PET/CT for [177Lu]LuPSMA therapy in metastatic prostate cancer (mCRPC)
Source: Cancer Imaging. 2026 Jun 30;26:84. doi: 10.1186/s40644-026-01066-0 (PMC13330063; doi:10.1186/s40644-026-01066-0)
Supplement: Supplementary file 1 — Supplementary Material 1 [file 40644_2026_1066_MOESM1_ESM.pdf]

Supplementary Material

Table S1: Quantitative Imaging Metrics. SUV and ADC metrics extracted at baseline and post-treatment for each target lesion.

	**Imaging Metric Details**
SUVmean	Mean SUV within the volume of interest (VOI)
SUVsd	Standard deviation of SUVmean within the VOI
SUVpeak	Mean SUV of a 1cm3 sub-volume contiguous to the voxel containing the highest uptake.
ADCmean	Mean ADC (mm2/s) within the VOI
ADCsd	Standard deviation of ADCmean (mm2/s) within the VOI
ADCkurtosis	Measure of tailedness in the ADC distribution
ADCvol	Anatomical volume derived from diffusion-weighted imaging (DWI) (ml)

Table S2: Effect of baseline predictors on odds of lesion-response in random effect logistic regression coefficients.

		Model 1		Model 2		Model 3		Model 4		Model 5
	**OR**	**95% CI**	**OR**	**95% CI**	**OR**	**95% CI**	**OR**	**95% CI**	**OR**	**95% CI**
Bone lesion	1.00	(reference)	1.00	(reference)	1.00	(reference)	1.00	(reference)	1.00	(reference)
Lymph node lesion	0.97	0.15, 6.37	2.25	0.18, 28.0	1.49	0.15, 14.74	3.60	0.27, 48.5	3.63	0.20, 65.3
SUVmean [1] (bone lesions)			1.50	1.10, 2.04					1.47	1.08, 2.01
SUVmean [1] (lymph node lesions)			1.18	0.97, 1.43					1.14	0.90, 1.45
ADCmean [2] (bone lesions)					1.17	0.83, 1.63				
ADCmean [2] (lymph node lesions)					0.66	0.39, 1.12				
log10(ADCvol)[3] (bone lesions)							4.26	0.68, 26.8	2.58	0.35, 19.2
log10(ADCvol) [3] (lymph node lesions)							6.23	0.58, 67.0	1.95	0.09, 42.2
σ_u	2.04	1.04, 3.99	2.36	1.13, 4.93	2.60	1.24, 5.43	2.24	1.07, 4.71	2.36	1.10, 5.09
Likelihood ratio test p-value (compared to model 1)			<0.001		0.124		0.060		0.002	
AIC	112.8		96.8		108.6		107.2		95.7	

Notes: [1] centred on 15; [2] centred on 900 then divided by 100; [3] centred on 0.4; σ_u: standard deviation of patient-level random effect (on log odds scale). AIC: Akaike Information Criterion.
